# Malnutrition and non-communicable diseases among Bangladeshi women: an urban–rural comparison

**DOI:** 10.1038/nutd.2017.2

**Published:** 2017-03-20

**Authors:** M S Zahangir, M M Hasan, A Richardson, S Tabassum

**Affiliations:** 1 Department of Statistics, University of Chittagong, Chittagong, Bangladesh; 2 Department of Mathematics & Statistics, University of Canberra, Bruce, ACT, Australia; 3 National Centre for Epidemiology & Population Health, Australian National University, Acton, ACT, Australia

## Abstract

**Background/Objectives:**

This study aims at examining the urban–rural differentials in the effects of socioeconomic predictors on underweight and obesity of ever-married women in Bangladesh. The effect of malnutrition and other risk factors on non-communicable diseases is also examined.

**Subjects/Methods:**

The information regarding nutritional status, socioeconomic and demographic background, and non-communicable diseases of ever-married women was extracted from the nationally representative, cross-sectional Bangladesh Demographic and Health Survey (BDHS 2011) data set. Both bivariate (*χ*^2^ test) and multivariate (multinomial logistic regression model) analyses were performed in determining the risk factors of malnutrition. The effect of malnutrition and associated risk factors on non-communicable diseases was determined using binary logistic regression models.

**Results:**

The overall prevalence as well as the effects of individual risk factors of malnutrition differ in urban and rural settings. Regional differentials in the prevalence of underweight were statistically significant only for rural areas. In rural and urban settings, women from households with poor economic status were 67% (odds ratio (OR) 0.33, 95% CI 0.26–0.43) and 81% (OR=0.19, 95% CI 0.13–0.29) less likely to be overweight, respectively, with respect to those from affluent households. Women from the Rangpur division were significantly more likely to suffer from anemia (OR=1.41, 95% CI 1.13–1.77) and hypertension (OR=1.67, 95% CI 1.19–2.34) than those from the Sylhet division (reference division). With respect to those considered as underweight, women who were categorized as overweight were 0.47 (OR=0.53, 95% CI 0.43–0.65) times less likely to suffer from anemia, and 1.83 (OR=2.83, 95% CI 1.99–4.02) and 1.70 (OR=2.70, 95% CI 2.09–3.50) times more likely to suffer from diabetes and hypertension, respectively.

**Conclusions:**

Rural–urban differentials in the effects of individual risk factors of malnutrition were observed. Wealth status of households and nutritional status of women showed significant effect on the prevalence of anemia, diabetes and hypertension.

## Introduction

Adequate nutrition is a precondition for attaining good health, maintaining quality life and accelerating national productivity.^1^ Alternatively, malnutrition appears as a threat for pregnant women, increases the susceptibility to diseases and accelerates maternal mortality ratios.^2, 3, 4^ Malnutrition can result in adverse pregnancy outcomes and underweight mothers are more likely to give births to babies with relatively lower weight.^5, 6, 7^ Such babies achieve poor psychological health^8^ and possess higher risks of mortality, and in the case of survival, higher risks of being underweight.^9, 10^ Owing to low dietary intake, inequitable distribution of food within a household, and poor food storage and preparation practices, malnutrition appears as a chronic problem among women in Bangladesh.^11, 12^ With 176 pregnancy-related deaths per 100 000 live births, Bangladesh is considered as a country with higher prevalence of maternal mortality ratios.^13^

On the other hand, rapidly increasing prevalence of obesity is becoming as a threat to the women from both developed and developing countries.^14, 15^ The World Health Organization estimated global prevalence of obesity as 400 million in 2005 and the predicted figure for 2015 was 700 million.^16^ Nationally representative surveys in Bangladesh, India and Nepal showed significant increases in the prevalence of obesity among women of reproductive age.^17, 18, 19^ In Bangladesh, the prevalence of obesity is increasing at an alarming rate (from 2.7 to 8.9% in the last 10 years) and the percentage of overweight women was considerably higher in urban areas (32.2%) than in rural areas (13.1%).^20^

The prevalence of nutritional deficiency was relatively higher among rural, illiterate and early married women and among those with a low standard of living.^18, 21^ Body mass index (BMI), an index of measuring the nutritional status, increases with increasing age, educational level of woman and her husband, household wealth, age at first marriage and age at first birth. Across most developing countries, higher prevalence of overweight was observed for higher educated women from the households categorized as higher wealth.^22^ Studies from India showed that the prevalence of obesity is more profound in women aged between 40 and 49 years.^23^ BMI has also been shown to decrease with increasing number of ever born children.^24^

A study^20^ on child and maternal nutrition in Bangladesh concluded that prevalence of chronic energy deficiency (BMI <18.5 kg m^−2^) in rural mothers is almost twice (27.7%) as large as the urban (15.9%) mothers. Although the prevalence of overweight has been widely acknowledged in urban areas, the burden is also substantial in rural areas.^25^

Nutrition also has interesting effects upon non-communicable diseases, such as anemia, diabetes and hypertension. The broad burden of non-communicable diseases in South Asia has been described,^26^ including diabetes and hypertension which are included in the present research, but also addressing cancer that is outside the scope of this research. Anemia has been the outcome focus of research in India,^27^ and in the intervening decade much more data have been collected both in India and neighboring countries such as Bangladesh, which is the focus of this research. Diabetes and its relationship to BMI among other factors has been a focus of the famous NHANES in the United States for many years.^28^ Other small-scale studies of risk factors for diabetes have taken place on the Indian subcontinent.^29^ Hypertension and its relationship to BMI has been studied in Pakistan^30^ but otherwise literature in this area appears to be scarce. Among women in Bangladesh, the prevalence of malnutrition and non-communicable diseases is increasing over the years. However, the rates, as well as the effect of covariates on the prevalence, may vary in urban and rural settings. The main rationale for this study lies in filling the gap existing in the literature in terms of comparing the effects of malnutrition and communicable diseases in urban–rural settings, using nationally representative data. This research aims to address that gap through two key objectives.

The principal research question of this paper is whether there are any urban–rural differentials in the effect of risk factors on prevalence of malnutrition (underweight and obesity) among women in Bangladesh. For the purpose, the effect of a wide range of potential covariates on nutritional status of women will be assessed using bivariate and multivariate analyses on both urban and rural settings. Considering the three categories of nutritional status (underweight, normal and overweight), the multivariable analyses will adopt multinomial logistic regression models. Secondly, the effect of malnutrition on non-communication diseases, such as anemia, diabetes and hypertension, will be assessed using binary logistic regression models. Models will be fitted to the data extracted from the nationally representative Bangladesh Demographic and Health Survey (BDHS 2011). The findings of this study can provide a comprehensive picture of nutrition and non-communicable disease for Bangladeshi women, which could inform and guide authorities in undertaking appropriate measures to improve health outcomes.

## Subjects and methods

### Source of data

The study was based on secondary data set obtained from nationally representative and cross-sectional Bangladesh Demographic and Health Survey conducted in 2011 (BDHS 2011). The DHS Program is approved by the ICF International Institutional Review Board and informed consent is obtained from all participants (see https://dhsprogram.com/What-We-Do/Protecting-the-Privacy-of-DHS-Survey-Respondents.cfm). The BDHS 2011 was based on a two-stage stratified sample of households. In the first stage, the country was divided into 20 strata, and using the probability proportional to size sampling technique, a sample of 600 (207 from urban, the rest from rural areas) Enumeration Areas (considered as the Primary Sampling Units or clusters) were selected independently from those strata. A complete list of households in the selected Enumeration Areas provided a sampling frame for the next stage. In the second stage, a systematic sample of 30 households was selected per cluster (Enumeration Areas) to provide a representative sample covering the seven administrative divisions. From the selected households, 18 222 ever-married women aged between 12 and 49 were identified, and the BDHS team interviewed 17 842 of them. This study considered a total of 16 026 respondents (11 832 from rural and 4194 from urban areas) who were not pregnant and did not give a birth in the preceding 2 months of the survey and provided complete information. Detailed information about the survey is available in the survey report.^31^ Information regarding non-communicable diseases was collected from women (and men) aged 35 or older from one-third of the previously selected households.

### Dependent and independent variables

The BMI (ratio of weight in kilograms to the square of height in meters), an indicator of nutritional status, is considered as the dependent variable in the study. For the current study, women were categorized as underweight (BMI up to 18.49 kg m^−^^2^), normal (BMI between 18.50 and 24.99 kg m^−^^2^) and overweight (BMI 25 kg m^−^^2^ or more).

Blood glucose was measured using the HemoCue 201+ blood glucose analyzer (HemoCue AB, Angelholm, Sweden) in capillary whole blood obtained from the middle or ring finger from adults after an overnight fast. The HemoCue 201+ analyzer displayed the blood glucose measurements in milligrams per deciliter (mg dl^−1^). This unit of measurement was converted into millimoles per litre (mmol l^−1^). The blood glucose measurements were adjusted to the plasma glucose equivalent values by multiplying each value by 1.11. Finally, based on plasma glucose level, women were categorized as normal (3.9–6.0 mmol l^−1^) or suffering from prediabetes (6.1–6.9 mmol l^−1^) or diabetes (7.0 mmol l^−1^ or more).

To determine the level of anemia among ever-married women, capillary blood was collected from a finger prick. The HemoCue system consists of a battery-operated photometer and a disposable microcuvette is used to measure hemoglobin (Hb) in blood as grams per deciliter (g dl^−1^). The prevalence of anemia is determined based on hemoglobin levels (adjusted for pregnancy status, altitude and smoking status). The subjects were categorized as anemic if the hemoglobin level is below 12 g dl^−1^ for non-pregnant and below 11 g dl^−1^ for pregnant women.

To measure blood pressure, the LIFE SOURCE UA-767 Plus Blood Pressure Monitor model (A&D Company Ltd, Tokyo, Japan) was used. Three measurements in millimeters of mercury (mm Hg) of both systolic pressure and diastolic pressure were taken during the survey at approximately 10-min intervals between measurements. The average of the second and third measurements was used to report respondent's blood pressure values. Women were considered as suffering from hypertension if the systolic pressure was more than 139 and diastolic pressure was more than 89.

The independent variables were chosen on the basis of existing literature and availability of data. The variables are related to the region (administrative divisions), background of respondent (education, access to media, age, age at first marriage, age at first birth, total children ever born and marital duration), background of partner (education) and the household (economic status) they live in. The independent variables used in the analysis were either based on the original coding or re-grouped into categories. The variable, access to media, was obtained as a combination of the variables, listen to radio, watch television and read newspaper. As an indicator of wealth, the DHS created a wealth score for each household in three steps. The first step involved selecting indicators common to urban and rural areas, such as household construction materials (roof, ceiling and floor), possessions (televisions and bicycles) and dwelling characteristics (source of drinking water, sanitation facilities). These categorical variables were then transformed into separate dichotomous indicators. Using the dichotomous indicators, in the second step, separate factor scores were produced for households in urban and rural areas. The separate area-specific (urban and rural) factor scores were then combined to produce wealth scores through a regression on the common factor scores. Detailed information about the calculation of the wealth scores is available in the survey report.^31^ This study adopted the wealth scores and categorized the households as poor (lowest 40%), middle (next 40%) and rich (upper 20%), the same groupings used by others.^32, 33, 34^ A list of independent variables, their levels and percentage distributions in both urban and rural settings is presented in Table 1.

### Methods

The BDHS 2011 adopted appropriate survey methodology to obtain a representative sample. However, the final sample does not guarantee complete representativeness in terms of proportional allocation at strata and cluster levels. To ensure the representativeness of the sample at various levels, sampling weights were calculated separately for each sampling stage and cluster based on sampling probabilities. The design of the survey (stratification and clustering) and sample weights were incorporated into the analyses using svy routines of STATA.^35^ The data are available upon application to the Demographic and Health Surveys Program (https://dhsprogram.com/). STATA code is available upon application to the corresponding author.

The dependent as well as all the independent variables considered in the study were categorical. Bivariate *χ*^2^ tests are performed to explore the association between individual predictor variables and nutritional status of women.^32, 33, 36^ Considering three categories of nutritional status (underweight, normal and overweight), the multinomial logistic regression technique was employed to estimate the net effect of covariates. In terms of non-communicable diseases, the women were categorized by the presence or absence of anemia, diabetes and hypertension. Binary logistic regression models were fitted to assess the effect of risk factors on non-communicable diseases. The effects of the predictor variables on dependent variables (nutritional level, anemia, diabetes and hypertension of ever-married women) were estimated by odds ratio (OR) of each category relative to the reference category. The OR of the reference category is 1. When the OR of a given category is greater than 1, it means higher odds of suffering from underweight, overweight, anemia, diabetes and hypertension compared with the reference category, and vice-versa. To compare the effect of predictors on malnutrition and obesity of women, separate analyses were performed for respondents from rural and urban areas. However, the models for the non-communicable diseases were fitted to the overall data set. Analysis on the data was performed using STATA (StataCorp LLC, College Station, TX, USA) and R (R Foundation for Statistical Computing, Vienna, Austria).^37^

## Results

### Overall prevalence

**Figure 1:**
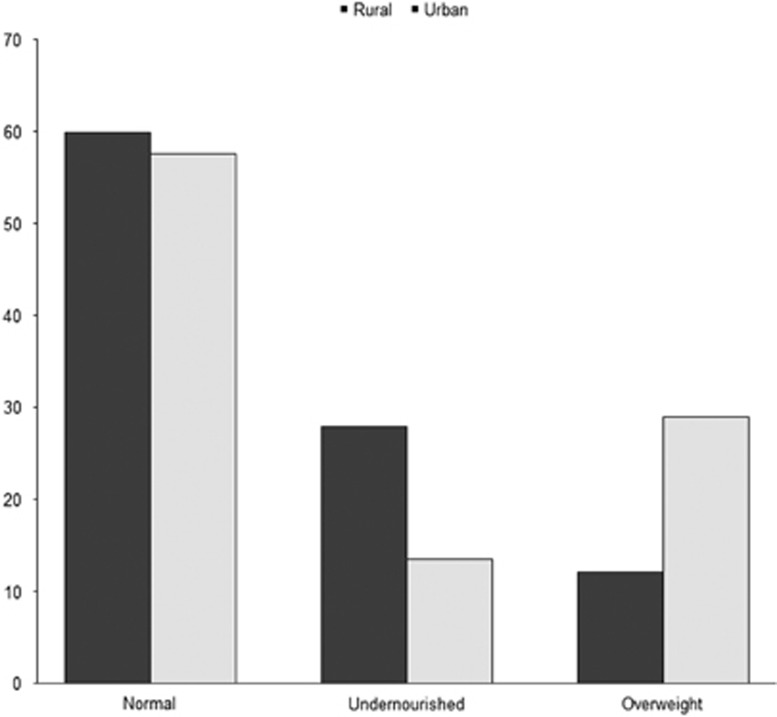
Percentages of women with various levels of nutrition and non-communicable diseases by place of residence. Confidence intervals (95%) not shown as width less than 0.001 in all cases.

**Figure 2:**
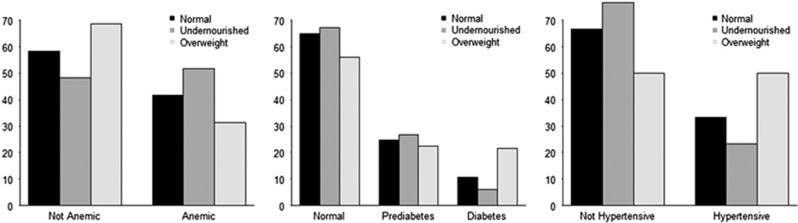
Percentages of women with various non-communicable diseases by nutritional status.

Prevalence of underweight was higher, and obesity was lower, for women residing in rural areas (Figure 1). The non-communicable diseases of hypertension and diabetes were most prevalent among overweight women, whereas anemia was most prevalent among underweight women (Figure 2).

### Bivariate analyses for nutrition

Table 2 represents the results from the tests of association between nutritional status of women and background characteristics. Significant associations existed between all the covariates and nutritional status of women from both rural and urban Bangladesh. In both settings, the prevalence of being underweight was the highest in Sylhet and of being obese was the lowest in Rangpur division. Only 8.83% of rural-illiterate women were obese, whereas the percentage was 41.43 for the urban-higher educated women. Percentages of being underweight among rural-illiterate and urban-higher educated women were 32.03 and 5.20, respectively. Partner's education (as considered as four levels) was also found to be crucial in determining the nutritional status of women. Household wealth had significant association with nutritional status of women and the association was more prominent in urban areas. Among the women from poor households, percentages of being underweight in rural and urban areas were 35.12 and 32.98, respectively. Percentages of overweight women were lower among poor (5.75 for rural and 6.90 in urban women) with respect to those from rich household (30.01 for rural and 39.57 for urban women). The percentages of underweight women were highest for women aged below 20 years (38.84 for rural and 24.29 for urban areas) and lowest for those aged between 31 and 40 years (23.67 for rural and 9.12 for urban areas). A huge difference in the percent of overweight women in the 31–40 year age group is observed in rural (15.68) and urban (38.50) areas. In both urban and rural settings, age at first marriage and age at first birth showed negative association with being underweight and positive association with overweight.

### Multivariable analyses for nutrition

The adjusted ORs of being underweight and overweight with confidence interval for various categories of predictor variables (with respect to the reference category) are presented in Table 3. Separate models are fitted for urban and rural areas. In this multivariable analysis, regional (division) differences in the prevalence of being underweight were more prominent in rural than in urban areas. However, regional variations in the prevalence of obesity of women were not statistically significant.

Except for underweight women in urban areas, the effect of education on nutritional status was insignificant. In urban areas, illiterate women and women with primary level of education were 1.75 (OR=2.75; CI=1.47–5.12) and 1.33 (OR=2.33; CI=1.27–4.28) times more likely to be underweight than those with above secondary education. In both urban and rural settings, household wealth showed significant effects of being underweight and overweight. In the rural setting, compared with women from affluent households, those from households categorized as poor and middle wealth were 1.89 (OR=2.89; CI=2.23–3.75) and 1.11 (OR=2.11; 1.65–2.70) times, respectively, more likely to be underweight. The figures for urban setting are 1.24 (OR=2.24; CI=1.55–3.24) and 0.32 (OR=1.32; CI=1.00–1.73), respectively. In urban setting, women from households in the poor and middle wealth categories were 81% (OR=0.19; CI=0.13–0.29) and 57% (OR=0.43; CI=0.35–0.53) less likely to suffer from overweight with respect to those from affluent households. In the multivariable setting, the effect of age of women, age at first marriage and age at first birth on nutrition status of women were found to be either inconsistent or statistically insignificant. In terms of total children ever born, women with no children were less likely to suffer from underweight and more likely to suffer from obesity.

### Multivariable analyses for non-communicable diseases

The adjusted ORs of non-communicable diseases with confidence intervals for various categories of predictor variables (with respect to the reference category) of ever-married women are presented in Table 4. Place of residence and geographical region show almost no association with disease status compared with the reference category. Household wealth does have a significant association with non-communicable disease, such that women from households in the middle and rich wealth categories were 18% (OR=0.82; CI=0.72–0.95) and 37% (OR=0.63; CI=0.52–0.77) less likely to suffer from anemia with respect to those from poor households. The association is reversed for diabetes, such that, with respect to women from poor households, those from households in the middle and rich wealth categories were 0.33 (OR=1.33; CI=0.97–1.82) and 1.66 (OR=2.66; CI=1.83–3.88) times more likely to suffer from diabetes. The same pattern holds for hypertension, where women from households in the middle and rich wealth categories were 0.08 (OR=1.08; CI=0.89–1.30) and 0.45 (OR=1.45; CI=1.15–1.84) times more likely to suffer from hypertension with respect to those from poor households. Finally, nutrition also has a significant association with non-communicable disease, such that women in normal or overweight categories were 28% (OR=0.72; CI=0.63–0.84) and 47% (OR=0.53; CI=0.43–0.65) less likely to suffer from anemia with respect to those who were underweight. The association is reversed for diabetes, such that women in normal or overweight categories were 0.61 (OR=1.61; CI=1.19–2.17) and 1.83 (OR=2.83; CI=1.99–4.02) times more likely to suffer from diabetes with respect to those who were underweight. The same pattern holds for hypertension, where women in normal or overweight categories were 0.52 (OR=1.52; CI=1.25–1.83) and up to 1.70 (OR=2.70; CI=2.09–3.50) times more likely to suffer from hypertension with respect to those who were underweight.

## Discussion

For the overall population, as well as for all background characteristics, higher percentages of underweight women and lower percentages of overweight women were observed for women from rural areas. The outcomes were consistent with studies elsewhere.^18, 21^ The observed lowest prevalence of being overweight for women from Rangpur, a division with the highest poverty rate, is expected. However, the highest prevalence of being overweight for women in Khulna, a division with a major agriculture based economy, is not expected.

In the multivariate analysis, regional effects on maternal nutrition were statistically significant in rural underweight women only. Educational status of their husband showed more significant effect on nutritional status of women than their own educational status. In both urban and rural settings, women from households categorized as poor or middle wealth were more likely to be underweight and less likely to be overweight. The fact is supported by other studies in Bangladesh.^21, 35^ This may reflect the fact that women from poor or middle wealth households may not afford sufficient foods to maintain their nutrition. Their lack of knowledge regarding relatively less expensive alternatives of balanced diet may result in adverse nutritional outcome. Alternatively, the higher proportion of overweight women from affluent households may be due to lack of physical activities and knowledge of healthy food habits. The major cities in Bangladesh are densely populated and there exists limited space for physical activity; moreover, junk food is becoming popular among the women of affluent households in those areas. These result in higher proportion of overweight women from affluent households residing in urban areas.

In terms of non-communicable diseases, underweight women were more likely to suffer from anemia and less likely to suffer from diabetes and hypertension. This may be due to the fact that both anemia and underweight share a common risk factor in terms of food intake. One study^30^ found an adjusted OR of 2.10 (CI=1.66–2.65) for diabetes and 2.32 (CI=2.00–2.69) for hypertension in the overweight group, figures comparable with the Bangladesh OR in this study of 2.83 (CI=1.99–4.02) and 2.70 (CI=2.09–3.50). Another study^29^ also found a significant association between BMI and diabetes in a rural population in Bangladesh (in the highest quintile of BMI, OR=2.15, CI=1.41–3.27). Regional differences also arise in the prevalence of non-communicable diseases, as seen in Table 4. In Rangpur, the prevalence of anemia and hypertension is higher, while in all other regions, the pattern of prevalence of diabetes and hypertension is similar.

There are several limitations in this study. Firstly, bias may be present due to misreporting of age, memory lapse and rounding in reporting of age. Paradoxical results have been found in the case of Khulna region with significantly lower risk of diabetes and higher risk of hypertension. Further in-depth study is required to identify the reason of the paradox. Secondly, the study utilized the largest cross-sectional survey conducted in Bangladesh in 2011. The BDHS is a large-scale survey covering a wide range of explanatory variables. Though the study uses the most recent data available (2011), the current situation with respect to maternal nutrition may have changed rapidly in the past five years.

In conclusion, bivariate and multivariate statistical analyses were performed to uncover the rural–urban differential in the prevalence of nutritional status and non-communicable diseases of ever-married women in Bangladesh. Rural–urban differentials were found everywhere, with increased levels of underweight across all background characteristics in rural areas compared with urban. On the other hand, in both rural and urban settings, wealth was found to have a consistent and significant effect on nutrition with poor women tending to be more likely to be underweight and less likely to be overweight than those from affluent households. The effect of nutrition on non-communicable diseases varies, with anemia prevalence rising with being underweight, whereas diabetes and hypertension prevalence rise with being overweight. Further research into regional differences in food security would be useful to identify if the result concerning the percentage of overweight women in Khulna and the prevalence of hypertension in Rangpur are reliable.

This paper has contributed to the advancement of knowledge in the realm of nutritional issues for women from the perspective of developing countries. The key factors influencing the prevalence of malnutrition and non-communicable diseases among women living in urban and rural areas have been identified. This study shows that the prevalence of underweight is much more profound among women living in rural areas. In both rural and urban settings, women from households categorized as poor are more likely to be underweight and to suffer from anemia. The main implication of this paper is that authorities should consider developing special programs emphasizing educating young women living in rural areas and urban slums regarding alternative low-cost balanced food options. This may help reduce underweight and anemia. Women living in urban areas, especially those aged over 30 years, are vulnerable to be overweight, and consequently to suffer from diabetes and hypertension. Targeted information campaigns should be introduced for women aged over 30 residing in urban areas to discourage the consumption of junk food and to encourage physical activity.

## Figures and Tables

**Table 1:** Percentage of rural and urban ever-married women distributed by selected background characteristics: 2011 BDHS

*Variables*	*Levels*	*Percentages of respondents*	
		*Rural (11 832)*	*Urban (4194)*
Geographical region	Barisal	6.1 (0.4)	3.5 (0.6)
	Chittagong	18.2 (0.7)	17 (1.1)
	Dhaka	25.9 (0.8)	50.2 (1.5)
	Khulna	13.2 (0.6)	10.2 (0.9)
	Rajshahi	16.8 (0.7)	10 (0.9)
	Rangpur	13.8 (0.6)	5.9 (0.7)
	Sylhet	5.9 (0.4)	3.2 (0.5)
Respondent's level of education	Illiterate	31.8 (0.8)	20.3 (1.2)
	Primary	31.6 (0.8)	25.7 (1.3)
	Secondary	32.4 (0.8)	38.9 (1.5)
	Higher	4.2 (0.4)	15.1 (1.1)
Partner's level of education	Illiterate	36.0 (0.9)	20 (1.2)
	Primary	28.1 (0.8)	23.4 (1.3)
	Secondary	26.4 (0.8)	31.6 (1.4)
	Higher	9.5 (0.5)	24.9 (1.3)
Respondent's access to mass media	No access	43.5 (0.9)	12.1 (1)
	Have access	56.5 (0.9)	87.9 (1)
Household wealth status	Poor	47.4 (0.9)	10.6 (0.9)
	Middle	43.4 (0.9)	33.6 (1.4)
	Rich	9.3 (0.5)	55.8 (1.5)
Age of respondent at interview	Up to 20 years	13.5 (0.6)	12.5 (1)
	21–30 years	37.1 (0.9)	37.7 (1.5)
	31–40 years	29.4 (0.8)	29.1 (1.4)
	41–49 years	20.1 (0.7)	20.6 (1.2)
Age of respondent at first marriage	Up to 15 years	43.8 (0.9)	35.3 (1.4)
	15–17 years	38.5 (0.9)	36.5 (1.5)
	18+ years	17.7 (0.7)	28.2 (1.4)
Age of respondent at first birth	Up to 16 years	38.2 (0.9)	31.4 (1.4)
	17–19 years	34.6 (0.9)	31.8 (1.4)
	20+ years	19.6 (0.7)	27.1 (1.3)
Total children ever born	No birth	7.5 (0.5)	9.7 (0.9)
	1–2 births	42.7(0.9)	52.2 (1.5)
	3–4 births	32.7 (0.8)	28.3 (1.4)
	5 + births	17.1 (0.7)	9.8 (0.9)
Marital duration	Up to 5 years	17.2 (0.7)	19.1 (1.2)
	6–13 years	27.9 (0.8)	29.5 (1.4)
	14–23 years	29 (0.8)	27.5 (1.4)
	24+ years	25.9 (0.8)	23.8 (1.3)

**Table 2:** Percentage of nutritional status for rural and urban ever-married women by background characteristics: 2011 BDHS

*Variables levels*	*Rural*			*Urban*		
	*Underweight*	*Normal*	*Overweight*	*Underweight*	*Normal*	*Overweight*
*Geographical region*						
Barisal	29.05	61.22	9.73	16.52	56.53	26.95
Chittagong	24.20	60.08	15.72	17.11	58.21	24.68
Dhaka	32.65	57.59	9.75	10.34	59.02	30.64
Khulna	20.07	63.67	16.26	15.09	52.13	32.78
Rajshahi	26.78	60.30	12.92	15.37	56.63	27.99
Rangpur	28.25	62.94	8.82	19.17	58.72	22.11
Sylhet	38.26	51.69	10.06	19.55	51.38	29.08
						
*Respondent's level of education*						
Illiterate	32.03	59.14	8.83	19.81	58.82	21.36
Primary	29.38	59.88	10.74	16.90	59.27	23.83
Secondary	24.55	60.29	15.16	11.22	57.47	31.31
Higher	13.17	63.01	23.76	5.20	53.37	41.43
						
*Partner's level of education*						
Illiterate	33.79	58.51	7.70	21.05	61.13	17.82
Primary	28.94	61.18	9.88	16.15	60.50	23.35
Secondary	23.67	60.13	16.21	12.93	58.88	28.19
Higher	14.98	60.92	24.10	5.71	50.36	43.93
						
*Respondent's access to mass media*						
No access	32.55	60.05	7.40	25.49	62.27	12.23
Have access	24.44	59.81	15.75	11.86	56.94	31.20
						
*Household wealth status*						
Poor	35.12	59.13	5.75	32.98	60.12	6.90
Middle	23.93	60.81	15.26	17.25	64.62	18.14
Rich	10.24	59.75	30.01	7.56	52.87	39.57
						
*Age of respondent at interview (years)*						
Up to 20	38.84	57.01	4.15	24.29	67.73	7.98
21–30	27.12	61.56	11.31	14.45	61.24	24.31
31–40	23.67	60.65	15.68	9.12	52.38	38.50
* *41–49	28.52	57.76	13.72	11.48	52.10	36.43
						
*Age of respondent at first marriage (years)*						
Up to 15	28.96	60.15	10.89	16.83	58.01	25.16
15–17	28.38	60.17	11.45	13.80	58.06	28.14
18+	24.62	58.77	16.61	8.99	56.46	34.55
						
*Age of respondent at first birth (years)*						
Up to 16	29.03	60.93	10.05	16.02	59.18	24.80
17–19	26.11	60.57	13.32	13.91	56.90	29.19
20+	27.36	57.69	14.95	8.71	53.77	37.52
						
*Total children ever born*						
0–2 births	27.92	60.07	12.01	13.22	59.38	27.40
3–4 births	26.11	60.74	13.15	11.60	54.82	33.58
5+ births	31.68	57.88	10.44	20.91	54.24	24.86
						
*Marital duration (years)*						
Up to 5	35.69	58.06	6.25	19.25	66.93	13.82
6–13	27.48	60.69	11.84	14.10	59.93	26.51
14–23	23.64	62.09	14.26	10.30	53.70	36.00
24+ years	28.20	57.88	13.92	11.90	52.36	35.74
Overall	27.97	59.91	12.12	13.51	57.59	28.90

**Table 3:** Odds ratios of nutritional status of rural and urban ever-married women by background characteristics: 2011 BDHS

*Background characteristics*	*Rural*		*Urban*	
	*Underweight*	*Overweight*	*Underweight*	*Overweight*
*Geographical region (RC Sylhet)*				
Barisal	0.64 (0.50–0.81)***	0.94 (0.67–1.32)	0.75 (0.48–1.74)	1.00 (0.71–1.41)
Chittagong	0.56 (0.45–0.69)***	1.28 (0.93–1.75)	0.70 (0.50–0.97)**	1.01 (0.72–1.42)
Dhaka	0.72 (0.59–0.88)***	0.96 (0.64–1.44)	0.53 (0.38–0.75)***	0.88 (0.63–1.22)
Khulna	0.45 (0.36–0.56)***	1.31 (0.95–1.80)	0.76 (0.54–1.07)	1.40 (0.94–2.11)
Rajshahi	0.61 (0.49–0.75)***	1.18 (0.86–1.64)	0.67 (0.46–0.98)**	1.11 (0.80–1.54)
Rangpur	0.57 (0.46–0.69)***	0.81 (0.58–1.15)	0.71(0.51–0.99)**	1.00 (0.70–1.44)
				
*Respondent's level of education* (*RC higher secondary)*				
Illiterate	1.47 (0.94–2.29)*	0.87 (0.58–1.29)	2.75(1.47–5.12)***	0.87 (0.62–1.22)
Primary	1.44 (0.94–2.19)*	0.91 (0.63–1.32)	2.33(1.27–4.28)***	0.97 (0.70–1.35)
Secondary	1.33 (0.88–2.00)	0.96 (0.69–1.34)	1.66 (0.95–2.87)*	1.04 (0.78–1.39)
				
*Partner's level of education (RC higher secondary)*				
Illiterate	1.58 (1.20–2.10)***	0.63(0.47–0.85)***	1.35(0.86–2.11)	0.58(0.41–0.82)***
Primary	1.37 (1.04–1.81)**	0.65(0.48–0.87)***	1.11(0.70–1.75)	0.72(0.53–0.97)**
Secondary	1.30 (1.00–1.71)*	0.90(0.69–1.16)	1.28(0.84–1.94)	0.69(0.55–0.85)***
				
*Respondent's access to mass media (RC have access)*				
No access	1.03 (0.91–1.16)	0.67 (0.57–0.79)***	1.12 (0.88–1.42)	0.67 (0.48–0.94)**
				
*Household wealth status (RC rich)*				
Poor	2.89 (2.23–3.75)***	0.33 (0.26–0.43)***	2.24 (1.55–3.24)***	0.19 (0.13–0.29)***
Middle	2.11 (1.65–2.70)***	0.64 (0.52–0.78)***	1.32 (1.00–1.73)**	0.43 (0.35–0.53)***
				
*Age of respondent at interview (RC 41–49)*				
Up to 20 years	1.59 (1.07–2.38)**	0.69 (0.38–1.26)	1.57(0.66–3.70)	0.47 (0.25–0.89)**
21–30 years	1.09 (0.81–1.47)	0.90 (0.61–1.33)	1.19 (0.55–2.58)	0.96 (0.63–1.46)
31–40 years	0.97 (0.79–1.18)	1.21 (0.95–1.55)	0.91 (0.52–1.59)	1.24 (0.94–1.65)
				
*Age of respondent at first marriage (RC>17)*				
<14	1.14 (0.90–1.44)	0.85 (0.66–1.11)	1.52 (1.04–2.21)**	0.98 (0.68–1.41)
15–17	1.10 (0.90–1.35)	0.82 (0.66–1.02)*	1.16 (0.81–1.66)	1.08 (0.79–1.48)
				
*Age of respondent at first birth (RC⩾20)*				
Up to 15 years	0.76 (0.64–0.91)***	0.94 (0.74–1.19)	0.80 (0.59–1.10)	0.85 (0.58–1.26)
15–17 years	0.75 (0.64–0.87)***	1.16 (0.94–1.43)	1.01 (0.77–1.33)	0.91 (0.70–1.19)
				
*Total children ever born (RC 5+)*				
0–2 births	0.76 (0.63–0.93)***	1.22 (0.96–1.54)	0.48 (0.34–0.69)***	1.08 (0.78–1.50)
3–4 births	0.85 (0.73–0.99)**	1.14 (0.91–1.43)	0.59 (0.43–0.80)***	1.08 (0.79–1.46)
				
*Marital duration* (RC⩾24)				
Up to 5 years	1.38 (0.93–2.03)	0.35 (0.21–0.61)***	2.33 (1.00–5.41)*	0.31 (0.17–0.55)***
6–13 years	1.04 (0.77–1.41)	0.65 (0.44–0.97)**	1.79 (0.81–3.94)	0.58 (0.36–0.94)**
14–23 years	0.84 (0.67–1.05)	0.77 (0.59–1.00)**	1.17 (0.64–2.11)	0.81 (0.57–1.16)

RC stands for reference category. Figures in parentheses indicate 95% confidence interval of odds ratio; ***P⩽0.01, **0.01<*P*⩽0.05, *0.05<*P*⩽0.10.

**Table 4:** Odds ratios of nutritional status of rural and urban ever-married women by background characteristics: 2011 BDHS

*Background characteristics*	*Anemia*	*Diabetes*	*Hypertension*
*Place of residence (RC rural)*			
Urban	0.93 (0.80–1.09)	1.05 (0.79–1.40)	1.03(0.84–1.27)
			
*Geographical region (RC Sylhet)*			
Barisal	1.25 (0.98–1.59)*	1.41 (0.91–2.20)	1.41 (1.03–1.93)**
Chittagong	1.01 (0.81–1.26)	1.19 (0.79–1.80)	1.09 (0.80–1.49)
Dhaka	1.28 (1.03–1.58)**	0.91 (0.61–1.35)	1.40 (1.04–1.89)**
Khulna	0.98 (0.79–1.23)	0.57 (0.36–0.91)**	1.55 (1.13–2.11)***
Rajshahi	1.21 (0.97–1.52)*	1.14 (0.74–1.76)	1.31 (0.96–1.78)*
Rangpur	1.41 (1.13–1.77)***	0.89 (0.54–1.48)	1.67 (1.19–2.34)***
			
*Household wealth status (RC Poor)*			
Middle	0.82 (0.72–0.95)***	1.33 (0.97–1.82)*	1.08 (0.89–1.30)
Rich	0.63 (0.52–0.77)***	2.66 (1.83–3.88)***	1.45 (1.15–1.84)***
			
*Nutrition (RC underweight)*			
Normal	0.72 (0.63–0.84)***	1.61 (1.19–2.17)***	1.52 (1.25–1.83)***
Overweight	0.53 (0.43–0.65)***	2.83 (1.99–4.02)***	2.70 (2.09–3.50)***

RC stands for reference category. Figures in parentheses indicate 95% confidence interval of odds ratio; ***P⩽0.01,**0.01<*P*⩽0.05,*0.05<*P*⩽0.10.
